# A Case of Lung Volume Reduction Surgery in a Young Adult With Chronic Lung Disease

**DOI:** 10.1002/rcr2.70303

**Published:** 2025-08-13

**Authors:** Hirohisa Horinouchi, Fumio Maitani, Jun Miyauchi

**Affiliations:** ^1^ Department of General Thoracic Surgery Saitama City Hospital Saitama Japan; ^2^ Department of Pathology Saitama City Hospital Saitama Japan

**Keywords:** chronic lung disease, chronic obstructive lung disease, emphysematous change, lung volume reduction surgery

## Abstract

Chronic lung disease, characterised by impaired development and/or fibrotic changes in the lungs of preterm neonates, results in lifelong consequences that affect respiratory well‐being. An 18‐year‐old male with chronic lung disease presented with shortness of breath upon exertion. Computed tomography revealed bilateral emphysema and a large bulla in the left lower lobe. Additionally, severe obstructive ventilatory impairment was observed and respiratory rehabilitation was ineffective in improving lung function. We performed lung volume reduction surgery on the left lower lobe, resulting in improved ventilatory function and exercise capability. This case shows that emphysematous changes in patients with chronic lung disease can be successfully treated with surgery.

## Graphical abstract

An 18‐year‐old boy with chronic lung disease reported experiencing shortness of breath during exertion for the past year. He was diagnosed with severe emphysema, along with a giant bulla in his left lower lobe. Treatment with bronchodilators and respiratory rehabilitation did not alleviate his symptoms. Consequently, he underwent lung volume reduction surgery on his left lower lobe. Following the procedure, his ventilatory function improved, and his symptoms were significantly reduced.

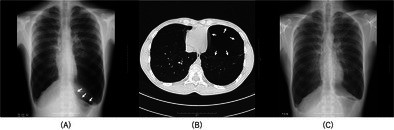

## 1 Introduction

Chronic lung disease in preterm neonates is characterised by impaired small airway development and fibrosis [1]. Clinical features include alveolar simplification, pulmonary fibrosis, chronic obstructive lung function, and susceptibility to infections [2, 3]. Furthermore, symptoms can persist into adulthood, becoming a lifelong burden [4]. We report the case of an 18‐year‐old male with chronic lung disease who underwent surgery for emphysematous changes.

## 2 Case Report

An 18‐year‐old male with chronic lung disease presented to a tertiary care hospital with a one‐year history of exertional shortness of breath. The patient was born at 28 weeks of gestation with an Apgar score of 1 at 5 min. He was subsequently diagnosed with hydrops fetalis, intubated, and transferred to the neonatal intensive care unit. The ventilator was removed after 8 weeks, followed by supplemental oxygen after 9 weeks. The patient was subsequently diagnosed with chronic lung disease and had been hospitalised several times throughout his infancy for pneumonia and asthma.

A physical examination at the time of his presentation revealed mild emaciation, with no skeletal deformities, and laboratory tests were normal. He could climb up two flight stairs with shortness of breath. Pulmonary function tests revealed obstructive and restrictive patterns. His vital capacity (VC) was 2.91 L (63.6%). The forced expiratory volume in 1 s (FEV1) was 1.05 L (%FEV1: 41.83%). Total lung capacity was 5.82 L (96.2%) but residual volume was 3.36 L (271%), indicating severe air trapping. DLCO showed a normal value of 35.37 mL/min/mmHg (133.9%).

**FIGURE 1:**
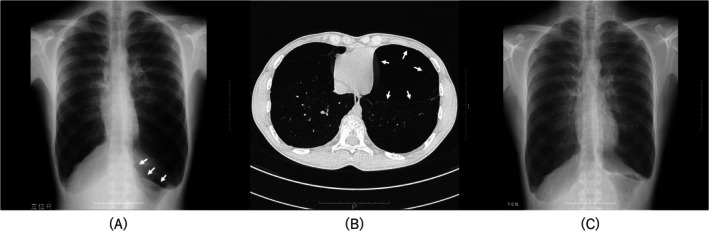
(A) Preoperative chest radiography showing bilateral lung hyperinflation and the depression of the left diaphragm (arrow) due to hyperinflation in the left lower lobe. (B) A chest radiography obtained 8 years postoperatively showing a physiological contour of the left diaphragm. (C) Preoperative computed tomography of the chest showing bilateral emphysematous lung parenchyma and a giant bulla in the left lower lobe.

Chest radiography showed bilateral lung hyperinflation and a reverse convex left diaphragm contour (Figure 1A). Computed tomography revealed heterogeneous emphysematous changes throughout the lungs with a large emphysematous bulla in the left lower lobe (Figure 1B).

**FIGURE 2:**
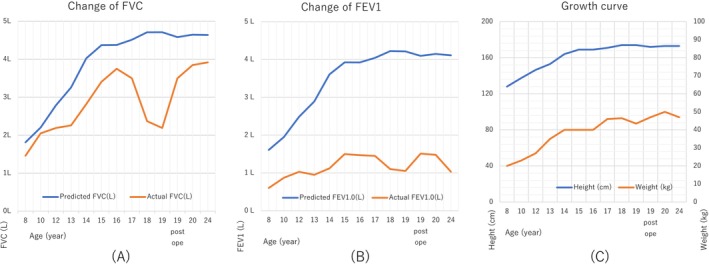
Change of spirometry and growth curve. (A) The patient's vital capacity (VC) reached a plateau at the age of 16, and began to decrease. VC recovered after surgery. (B) Forced expiratory volume in 1 s (FEV1) also increased with age, although the value was lower than the normal value. FEV1 recovered after surgery. (C) Growth curve of height (cm) and weight (kg).

Respiratory rehabilitation and steroid‐free bronchodilators were initiated; however, the patient's ventilatory function did not improve (VC, 2.79 L; FEV1, 1.01 L; Figure 2). Therefore, we planned lung volume reduction surgery (LVRS) to improve his ventilation mechanics.

After providing informed consent, the patient underwent video‐assisted thoracoscopic LVRS; the boundary between the bulla and normal lung parenchyma was not clearly defined. Volume reduction was performed using reinforced staplers (Tri‐Staple 2.0, Reinforced Reload; Medtronic, Minneapolis MN, USA), with a fibrin sealant and a polyglycolic acid sheet to cover the staple line.

On postoperative Day 2, the chest tube was removed after the absence of air leakage was confirmed; however, on postoperative Day 3, the patient developed a massive pneumomediastinum and subcutaneous emphysema after a bowel movement. Although computed tomography revealed no pneumothorax, the chest tube was reinserted, and supplemental nasal oxygen (2 L/min) was administered. By postoperative Day 9, the pneumomediastinum and subcutaneous emphysema had resolved, allowing for the chest tube and supplemental oxygen to be removed. The patient was discharged on postoperative Day 12.

Histologically, typical emphysema was observed. The terminal bronchioles showed a sparse layer of smooth muscle, indicative of impaired lung development in premature infants. The pulmonary arteries did not exhibit hypertrophic intimal changes or muscular hypertrophy.

Significant improvement was observed both clinically and in lung function (Figure 2), and 3 years after surgery, bronchodilators were discontinued upon the patient's request.

A chest radiograph obtained 8 years post‐surgery showed the physiological contour of the diaphragm (Figure 1C), and although the patient's FEV1 had decreased slightly, he had no shortness of breath. At 9 years post‐surgery, the patient's exercise limitations were minimal.

## 3 Discussion

Various studies have analysed the evolution of lung function in young adults with chronic lung disease, highlighting persistent small airway obstruction, hyperinflation, and hypersensitivity [5, 6, 7].

This patient's complaint of dyspnea developed at 17 years of age, with progressive deterioration linked to the development of emphysema and expansion of an emphysematous bulla. His pulmonary function was assessed throughout the course of the disease. During infancy and adolescence, both the VC and FEV1 increased annually, although they remained consistently below age‐adjusted norms.

Baraldi et al. [8] noted that improvement in lung function among patients with chronic lung disease, with a similar growth peak, was related to age, although ventilatory indices remained low. Their work further clarified the early onset of declining lung ventilatory function in patients with chronic lung disease and confirmed that hyperinflation and chronic obstructive changes were key features in the development of bronchopulmonary dysplasia [8, 9].

Our patient's VC recovered significantly post‐surgery, reaching preoperative levels. FEV1 also showed some recovery, although the actual increase was not substantial. The surgery‐corrected ventilation mechanics did not alter the physiology of the small airways (Figure 2). Long‐term observations revealed a secondary decline in the patient's ventilation function. This decline might have been partly due to the discontinuation of the daily bronchodilators; however, it is also possible that the progression of emphysematous changes may have contributed to the decrease in FEV1.

According to the National Emphysema Treatment Trial study, LVRS was beneficial for patients with emphysema, especially those whose lungs were affected in the upper lobe [10]. The key to a successful LVRS is the reduction of the attenuation area. In our patient, the primary attenuation areas with severe emphysema and bullous changes were located in the left lower lobe; therefore, the bullectomy/LVRS of the left lower lobe improved his ventilatory mechanics.

Dani et al. [11] reported the results of double lung transplantations in 32 patients with chronic lung diseases who underwent transplant surgery between 2000 and 2020, with a 5‐year survival rate of 81% [11]. As candidates for lung transplantation are limited, LVRS may be an alternative approach for patients whose lungs are compromised due to chronic lung disease.

## Author Contributions

Hirohisa Horinouchi and Fumio Maitani contributed to the design and implementation of the case report. Hirohisa Horinouchi prepared the manuscript. Fumio Maitani reviewed the manuscript. Jun Miyauchi interpreted pathological slides. All of the authors discussed the study and contributed to and approved the final manuscript.

## Consent

The authors declare that the patient provided written informed consent for the publication of this manuscript and the accompanying images.

## Conflicts of Interest

The authors declare no conflicts of interest.

## Data Availability

The data that support the findings of this study are available from the corresponding author upon reasonable request.

## References

[1] B. Stoecklin , S. J. Simpson , and J. Jane Pillow , “Bronchopulmonary Dysplasia: Rationale for a Pathophysiological Rather Than Treatment Based Approach to Diagnosis,” Pediatric Respiratory Review 32 (2019): 91–97. 10.1016/j.prrv.2018.12.002 30745153

[2] W. H. Northway, Jr. , R. B. Moss , K. B. Carlisle , et al., “Late Pulmonary Sequelae of Bronchopulmonary Dysplasia,” New England Journal of Medicine 323, no. 26 (1990): 1793–1799, 10.1056/NEJM199012273232603. 2247118

[3] T. Halvorsen , B. T. Skadberg , G. E. Eide , O. D. Røksund , K. H. Carlsen , and P. Bakke , “Pulmonary Outcome in Adolescents of Extreme Preterm Birth: A Regional Cohort Study,” Acta Paediatrica 93, no. 10 (2004): 1294–1300. 15499947

[4] A. Bhandari and S. McGrath‐Morrow , “Long‐Term Pulmonary Outcomes of Patients With Bronchopulmonary Dysplasia,” Seminars in Perinatology 37, no. 2 (2013): 132–137, 10.1053/j.semperi.2013.01.010. 23582968

[5] L. W. Doyle , E. Carse , A. M. Adams , S. Ranganathan , G. Opie , and J. L. Y. Cheong , “Victorian Infant Collaborative Study Group. Ventilation in Extremely Preterm Infants and Respiratory Function at 8 Years,” New England Journal of Medicine 377, no. 4 (2017): 329–337, 10.1056/NEJMoa1700827. 28745986

[6] S. J. Simpson , L. Turkovic , A. C. Wilson , et al., “Lung Function Trajectories Throughout Childhood in Survivors of Very Preterm Birth: A Longitudinal Cohort Study,” Lancet Child Adolesc Health 2, no. 5 (2018): 350–359, 10.1016/S2352-4642(18)30064-6. 30169268

[7] A. M. Gibson , C. Reddington , L. McBride , C. Callanan , C. Robertson , and L. W. Doyle , “Lung Function in Adult Survivors of Very Low Birth Weight, With and Without Bronchopulmonary Dysplasia,” Pediatric Pulmonology 50, no. 10 (2015): 987–994, 10.1002/ppul.23093. 25195792

[8] E. Baraldi and M. Filippone , “Chronic Lung Disease After Premature Birth,” New England Journal of Medicine 357, no. 19 (2007): 1946–1955, 10.1056/NEJMra067279. 17989387

[9] J. L. Hankinson , J. R. Odencrantz , and K. B. Fedan , “Spirometric Reference Values From a Sample of the General U.S. Population,” American Journal of Respiratory and Critical Care Medicine 159, no. 1 (1999): 179–187, 10.1164/ajrccm.159.1.9712108. 9872837

[10] K. S. Naunheim , D. E. Wood , Z. Mohsenifar , et al., “National Emphysema Treatment Trial Research Group. Long‐Term Follow‐Up of Patients Receiving Lung‐Volume‐Reduction Surgery Versus Medical Therapy for Severe Emphysema by the National Emphysema Treatment Trial Research Group,” Annals of Thoracic Surgery 82, no. 2 (2006): 431–443, 10.1016/j.athoracsur.2006.05.069. 16888872

[11] A. Dani , D. Hayes, Jr. , A. Guzman‐Gomez , et al., “Lung Transplantation for Bronchopulmonary Dysplasia,” Chest 163, no. 23 (2023): 032, 10.1016/j.chest.2022.12.032. PMC10206512 36610665

